# Transgene Delivery to Human Induced Pluripotent Stem Cells Using Nanoparticles

**DOI:** 10.3390/ph14040334

**Published:** 2021-04-06

**Authors:** Megan A. Yamoah, Phung N. Thai, Xiao-Dong Zhang

**Affiliations:** 1 Department of Economics, University of Oxford, Oxford OX1 3UQ, UK; yamoah.megan@gmail.com; 2 Department of Internal Medicine, School of Medicine, University of California, Davis, CA 95616, USA; pnthai@ucdavis.edu; 3 Department of Veterans Affairs, Northern California Health Care System, Mather, CA 95655, USA

**Keywords:** nanoparticles, magnetic nanoparticles, human induced pluripotent stem cells, human induced pluripotent stem cells-derived cardiomyocytes, transgene, transfection, nanomedicine, SARS-CoV-2, COVID-19, vaccine

## Abstract

Human induced pluripotent stem cells (hiPSCs) and hiPSCs-derived cells have the potential to revolutionize regenerative and precision medicine. Genetically reprograming somatic cells to generate hiPSCs and genetic modification of hiPSCs are considered the key procedures for the study and application of hiPSCs. However, there are significant technical challenges for transgene delivery into somatic cells and hiPSCs since these cells are known to be difficult to transfect. The existing methods, such as viral transduction and chemical transfection, may introduce significant alternations to hiPSC culture which affect the potency, purity, consistency, safety, and functional capacity of hiPSCs. Therefore, generation and genetic modification of hiPSCs through non-viral approaches are necessary and desirable. Nanotechnology has revolutionized fields from astrophysics to biology over the past two decades. Increasingly, nanoparticles have been used in biomedicine as powerful tools for transgene and drug delivery, imaging, diagnostics, and therapeutics. The most successful example is the recent development of SARS-CoV-2 vaccines at warp speed to combat the 2019 coronavirus disease (COVID-19), which brought nanoparticles to the center stage of biomedicine and demonstrated the efficient nanoparticle-mediated transgene delivery into human body. Nanoparticles have the potential to facilitate the transgene delivery into the hiPSCs and offer a simple and robust approach. Nanoparticle-mediated transgene delivery has significant advantages over other methods, such as high efficiency, low cytotoxicity, biodegradability, low cost, directional and distal controllability, efficient in vivo applications, and lack of immune responses. Our recent study using magnetic nanoparticles for transfection of hiPSCs provided an example of the successful applications, supporting the potential roles of nanoparticles in hiPSC biology. This review discusses the principle, applications, and significance of nanoparticles in the transgene delivery to hiPSCs and their successful application in the development of COVID-19 vaccines.

## 1. Introduction

Induced pluripotent stem cells (iPSCs) are derived from somatic cells that are genetically reprogrammed into an embryonic-like, pluripotent state capable of differentiating into all three germ layers. Since the landmark study reporting iPSCs [1], the field has greatly expanded [2,3,4,5,6]. Human iPSCs (hiPSCs) have the potential to revolutionize regenerative and precision medicine by providing a limitless source of differentiated cells for biomedical research, cell-based therapy, disease modeling, drug testing, and high-throughput drug discovery in a patient-specific manner [4,5,7,8].

**Figure 1:**
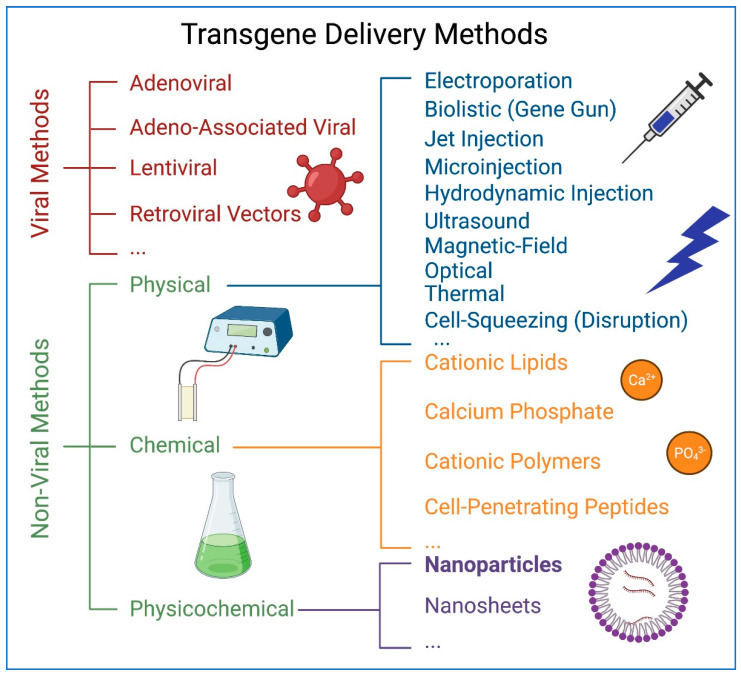
The summary and category of transgene delivery methods. Figure generated using BioRender.

Generation and genetic modification of hiPSCs represent the essential procedures for the study and application of iPSCs. The methods and platforms for reprogramming somatic cells to generate iPSCs have been evolving in the past decade [6,9,10]. For scientific studies and biomedical applications, expression of different tracking markers and transcription factors in human iPSCs (hiPSCs) is required. With the advent of genome editing using CRISPR-Cas9 technology [11,12], it has become increasingly feasible to correct disease-causing mutations in patient-specific hiPSCs in order to create isogenic lines for disease modeling or potential therapeutics [13,14]. However, there are significant technical challenges for transgene delivery into hiPSCs since these cells are notoriously difficult to transfect [15,16,17,18]. Currently, the major transgene delivery methods are commonly divided into two categories: viral and non-viral methods, and the non-viral method is further subdivided into chemical, physical, and physicochemical methods (Figure 1). Viral transgene delivery method has been widely used with high transduction efficiency and capacity of long-term transgene expression. Four types of viral vectors are widely used in transgene delivery, including adenoviral, adeno-associated viral, lentiviral, and retroviral vectors. However, the usage of viral transduction raises safety concerns including cytotoxicity, cellular immune responses, transgene integration into host genome, and generation of nonspecific mutations. Non-viral methods, including physical methods such as injection or electroporation and chemical methods such as lipofection, are potentially safer alternatives for transgene delivery into hiPSCs. However, their transfection efficiency is relatively lower [16,17]. Therefore, although multiple methods of genetic modifications exist, their efficiency, cytotoxicity, safety, and cost remain unsatisfactory [15,18]. Increasingly, nanoparticles have been used in biological research and medicine as powerful tools for transgene and drug delivery, imaging, diagnostics, and therapeutics [19,20]. A very successful application of nanoparticles in biomedicine is the extreme fast-pace development of SARS-CoV-2 vaccines, which has saved lives and has helped people to resist the challenges of the COVID-19 pandemic [21,22,23,24,25,26,27]. Nanoparticle-mediated transgene delivery method integrates the strategies and advantages of both the chemical and physical methods, demonstrating the promising and huge application potential. We therefore put it in the physicochemical transfection category (Figure 1). This review will discuss principles and applications of nanoparticles in transgene delivery.

## 2. Physical, Chemical, and Biological Properties of Nanoparticles

Over the past two decades, nanotechnology has revolutionized the fields from astrophysics, semiconductor industry to biology, as evidenced in the rise of nanotechnology-focused publications and the continued investment in nanotechnology research through the National Nanotechnology Initiative [28]. Since the 1950s, small-scale technologies slowly made their way into the semiconductor industry, first through reducing the size of transistors and then through advancing toward molecular electronics [29]. Today, the semiconductor industry has embraced new forms of nanoscale technology in two-dimensional materials and flexible circuit boards.

Nanoparticles are microscopic structures with at least one dimension less than 100 nm, which subsequently demonstrate novel physical and chemical properties from their bulk materials [30,31]. The most important properties on a nanoscale is the increased surface area to volume ratio. The increasing surface area to volume ratio leads to an increase in the dominance of the surface atoms of the nanoparticle over those in its interior [30]. The shape of the nanoparticles varies from spherical to a variety of other shapes such as cube, prism, hexagon, octahedron, disk, wire, rod, tube, etc. Based on the properties of composite materials, nanoparticles can be classified into simple and core/shell (composite) nanoparticles [30]. Simple nanoparticles are made from one material. Whereas, core/shell particles are made of two or more materials. The core/shell type nanoparticles consist of a core (inner material) and a shell (outer layer material). The material combinations of core and shell include inorganic/inorganic, inorganic/organic, organic/inorganic, and organic/organic materials [30]. The choice of shell material is critical and strongly dependent on the end application purpose. During the production process, several properties of nanoparticles such as dimensions, uniformity, surface area, and superparamagnetism can be well controlled for specific applications. At room temperature, using the simple “particle in a box” model from quantum mechanics, we expect to see quantum phenomena arise for systems a few nanometers in length. At these scales, classical conceptions of physics become less able to describe physical phenomena. It is also around these scales that nanotechnologies, such as quantum dots and nanoparticles, operate.

## 3. Nanoparticles in Biomedicine

While molecular electronics has yet to truly take hold in the electronics industry, the rise of nanotechnology has brought new-found applications in the field of biomedicine. The major classes of nanoparticles in biomedical applications include lipid-based nanoparticles, inorganic nanoparticles and polymeric nanoparticles. Lipid-based nanoparticles are organic nanoparticles with various structures, typically spherical with lipid bilayer wrapping at least one internal aqueous compartment, and classically exemplified as liposomes. Lipid-based nanoparticles have many advantages including formulation simplicity, less toxicity for in vivo application, biocompatibility, high bioavailability, larger payloads, and a range of physicochemical properties that can be controlled to modulate their biological characteristics [32]. Therefore, lipid-based nanoparticles are the most common class of FDA-approved nanomedicines [33]. Lipid-based nanoparticles are typically composed of phospholipids, which are the major components of biological membranes. Inorganic nanoparticles are usually made of inorganic materials, which can be precisely formulated with a variety of sizes and surface areas, structures and shapes. Inorganic nanoparticles have unique physical, electrical, magnetic and optical properties due to the properties of the core material. The coatings of the inorganic nanoparticles can be modified to define the surface properties for multiple biomedical applications. Iron oxide is a commonly used material for inorganic nanoparticles synthesis, and iron oxide nanoparticles make up the majority of FDA-approved inorganic nanomedicines [32,34]. Polymeric nanoparticles are organic nanoparticles usually synthesized by natural or synthetic materials, as well as monomers or preformed polymers with a variety of structures and properties. The most common types of polymeric nanoparticles are nanocapsules and nanospheres. Within these two large categories, polymeric nanoparticles are further divided into polymersomes, micelles, and dendrimers based on shapes [32]. The inorganic nanoparticles are usually coated (core/shell or composite) by polymers to gain the surface properties in biomedical application.

**Figure 2:**
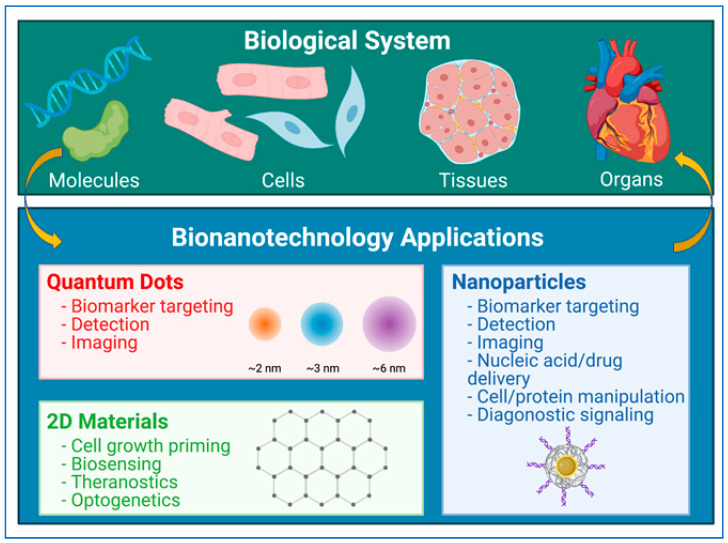
Bionanotechnology applications in biological system. Various applications of nanotechnology in biological research and medicine. Figure generated using BioRender.

As outlined in Figure 2, nanotechnology has wide-spread applications in biological system such as biomarkers, imaging, diagnostics, and therapeutics. Advances in medical imaging through nanomaterial-based technologies have led to improved results in optical, nuclear, ultrasound, and magnetic resonance imaging (MRI) [35,36,37]. Some of the earliest advances made use of paramagnetic iron oxide particles to image cancerous tissue in lymph nodes [38]. More recent applications have drawn from novel quantum physics structures, such as quantum dots [39,40], liposomes [41], nano-shells [42], and nanotubes [40,43] to target biomarkers. These technologies allow for the targeting of various structures and tissues for imaging purposes, greatly improving the accuracy of diagnoses.

Nanoparticles have also found place in nucleic acid and drug delivery and nano-therapeutics. The nanoparticles used in drug delivery include protein-drug conjugated, polymer-based, liposomal, and dendrimer-based [44]. Nucleic acids and drugs can be physically or chemically conjugated to the polymer for delivery. This facilitates delivery of various payloads including hydrophobic and hydrophilic compounds, as well as cargos with different molecular weights such as small molecules, macromolecules including nucleic acids, proteins and vaccines. These applications make polymeric nanoparticles ideal for delivery applications in biological system. By modulating properties such as composition, stability, responsivity, and surface charge of the polymeric nanoparticles, the loading efficacies and release kinetics of these therapeutics can be precisely controlled [32]. Targeting techniques for cancer treatments [44] include passive targeting reliant on passive accumulation [45], active targeting using receptors [46], or triggered targeting through pH-based [47] or external stimuli such as UV-light [48], ultrasound [49], or electric and magnetic field [50]. Similar techniques have also been applied for neurological and cardiovascular disorders [51].

With advances in nanotechnology and nanoparticle production techniques, the availability and flexibility of nanoparticle platforms have significantly increased. Lipid-based nanoparticles have been employed in the novel mRNA vaccines as those recently made against the SARS-Cov2 coronavirus [21,22,23,24,25,26,27]. These vaccines employ lipid-based nanoparticle capsules which carry the mRNA for coding the spike protein of the virus. The biological applications of nanoparticles continue to increase quickly as manufacturing techniques improve.

New technologies based on nanotechnology have been transformed into numerous biological applications, and the use of nanoparticles for targeted delivery has undoubtedly improved drug delivery capabilities. Magnetic nanoparticles are of particular interest due to their ability to be drawn to specific tissues reliably and with little disruption to the tissue itself [52,53,54,55,56]. As such, they represent an important branch within nanotechnology with significant potential for application, for example, in transfection of hiPSCs.

## 4. Nanoparticles in the Development of COVID-19 Vaccines

Since the emergence of the 2019 coronavirus disease (COVID-19) caused by the severe acute respiratory syndrome coronavirus 2 (SARS-CoV-2), there have been approximately 115 million confirmed cases globally and about 2.6 million global deaths as of 4 March 2021 [57]. Consequently, and expectedly, vaccine development has since accelerated as more structural information about SARS-CoV-2 and its variants becomes available. Currently, the four major structural proteins that may produce an immune response are the spike protein, nucleocapsid protein, membrane protein, and the envelope protein [58,59,60]. The most common target is the SARS-CoV-2 spike protein, which mediates cellular entry via the angiotensin-converting enzyme 2 (ACE2) cell receptor [61]. As rapid development of vaccines continues, the practicality and versatility of nanoparticles in COVID-19 vaccine formulation become more apparent [27,62]. In contrast to previously conventional vaccines that took advantage of live-attenuated, inactivated, or killed forms of a particular pathogen, these nanoparticle-based vaccines may be synthetic or virus-like particles (VLPs) that encapsulate nucleic acids or antigens or present conjugated-antigens on the surface [27]. Additionally, nanoparticles may be engineered to perform target-specific deliveries to antigen presenting cells (APCs) at specific regions in the body [63,64] and may even serve as adjuvants to accentuate immunogenicity by producing more robust cellular and humoral immunity [65].

**Figure 3:**
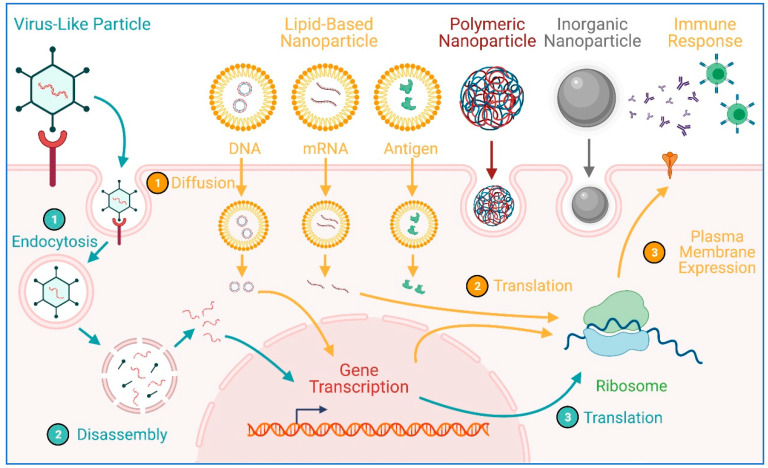
Potential applications of nanoparticles in the development of COVID-19 vaccines. Figure generated using BioRender.

Pertinent to the pandemic, the utilization of nanoparticles/nanocarriers has made headlines, as BioNTech/Pfizer [66] and Moderna [67] both developed mRNA COVID-19 vaccines using lipid-based nanoparticles [23], which demonstrated 94–95% efficacy in clinical trials [68]. These nanoparticles ensure effective delivery of mRNA into cells where they get translated into the spike protein that would subsequently trigger an immune reaction. Lipid-based nanoparticles, however, are just one of a few broad categories of nanoparticle delivery platforms that have been used for COVID-19 vaccine development. In addition to lipid-based delivery vehicles like liposomes, polymeric (e.g., polylactide, chitosan), metal and nonmetal inorganic (e.g., gold, silica, silver), and VLPs nanoparticles may be engineered and exploited (Figure 3) [27,62,69]. Indeed, the most recently approved COVID-19 vaccine in the United States is the Johnson & Johnson (J&J) vaccine, which utilizes an adenovirus that carries a SARS-CoV-2 DNA particle that codes for the spike protein [70]. Despite the relatively low 66% overall efficacy of the J&J vaccine, it is relatively stable at room temperature and does not require ultracold storage conditions like the BioNTech/Pfizer and Moderna vaccines, which makes vaccine distribution to underprivileged and underdeveloped areas more feasible. These three FDA-approved COVID-19 vaccines are not the only ones that have used nanoparticle technology. Many COVID-19 vaccine candidates from all around the world to some extent have employed nanoparticles, as they operate on the same size scale [25,27]. The rapid development and subsequent FDA approval in record time have illuminated the great potential for nanoparticle technology in vaccine development. Indeed, the challenges of the pandemic have presented an opportunity to refine this wonderful technology.

## 5. Magnetic Nanoparticles

The application of magnetic particles in medicine can be dated back to the 1950s when Gilchrist et al. used a magnetic field-heated metallic particles to destroy metastasis in lymph nodes missed at operation [71]. Magnetic particles were progressively used as the supportive materials and tools in many applications including enzyme immobilization, drug delivery, and cell separation [54,72,73,74]. Most materials that we consider “magnetic” in fact exhibit a specific type of magnetism, called ferromagnetism. In these materials, the internal magnetic dipoles are aligned and thus react, in bulk, to magnetic fields. Other types of materials, such as paramagnetic materials, also interact with magnetic fields, though much more weakly than ferromagnetic materials [75,76]. Magnetic fields interact with paramagnetic materials by inducing internal magnetic fields. Both ferromagnetic and paramagnetic materials are then attracted to the poles of the field, just as iron filings are attracted to the two ends of a bar magnet. Magnetic nanoparticles are characterized by their response to external magnetic field and their size of around a hundred nanometers [75]. They usually have an inorganic iron oxide core with organic or inorganic shell or coating. Their size means that, at room temperature and even less so at body temperatures, they tend not to exhibit quantum behavior. However, they also become extremely useful for applications on the cellular level.

The essential magnetic properties of nanoparticles offer potential application possibilities which are of great biomedical interest [77]. Magnetic nanoparticles have application in in vivo imaging, targeted drug delivery, transgene delivery, and image guided diagnosis and therapy [52,53,54,55,56,75,77]. They were also used in a range of cells, from cancerous cells to hiPSCs and hiPSC-derived cells. Specifically, they have been successfully used to deliver transgenes to hiPSC-derived cardiomyocytes [78]. Here, magnetic nanoparticles serve as an essential alternative for hiPSC transfections due to higher efficiency and better targeting. In all cases, the magnetic quality of these nanoparticles facilitates control and detection through magnetic fields.

**Figure 4:**
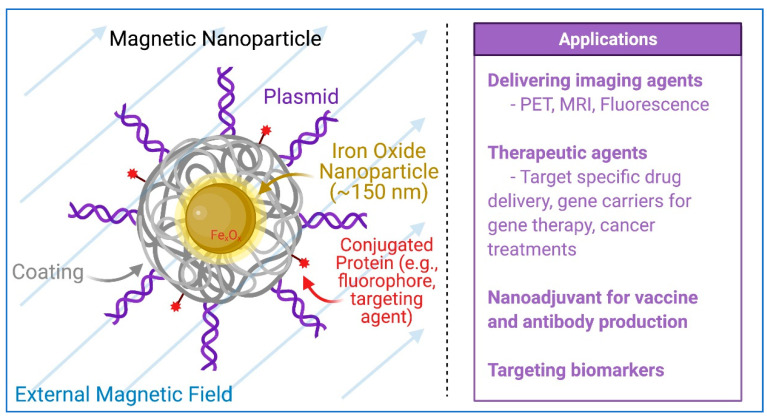
Iron oxide nanoparticles and applications. Iron oxide nanoparticle under an external magnetic field (light blue lines) with internal magnetic diploe aligned. For biological applications, nanoparticles are coated with an inorganic or organic coating, often to bind or contain other molecules for biological use. Figure generated using BioRender.

The most common nanoparticle used for drug delivery applications are superparamagnetic iron oxide nanoparticles [79]. They exhibit a specific type of paramagnetism, called superparamagnetism, which occurs for small particles of ferromagnetic materials. Superparamagnetic nanoparticles around 10–100 nm in size exhibit strong magnetic behavior under a magnetic field and act as a single magnetic domain. As shown in Figure 4, the internal magnetic field of superparamagnetic nanoparticles fully align, as one domain, with external magnetic fields. However, in the absence of a magnetic field, they do not produce their own field. For biological applications, superparamagnetic nanoparticles are given an inorganic or organic coating, onto which the drug or nucleic acid is loaded [78,79]. For example, for the transfection of hiPSCs, a superparamagnetic iron oxide nanoparticle is usually coated with specific proprietary cationic molecules with which transgenes can be associated. The transgene-bond magnetic nanoparticles can be magnetized, directed to, and concentrated on the cell surface using a magnetic plate. A similar technique can be used to direct nanoparticles in three-dimensional space using advance magnetic field techniques. These particles have already proved useful for targeted imaging techniques based on MRI technology [80]. Similar strategies were used for directed drug delivery [81].

## 6. HiPSCs and hiPSC-Derived Cardiomyocytes (hiPSC-CMs)

iPSCs came to the center stage in stem cell biology and regenerative medicine since the original publication and description of iPSCs [1]. HiPSCs are emerging as a promising strategy with potentials to revolutionize tissue engineering, industrial and personalized drug testing and screening, human disease modeling and treatment, and precision medicine [5,82]. HiPSCs are of human origin carrying complete human genomes, and they are pluripotent, a potential to be differentiated into any types of human somatic cell types. HiPSCs are stems cells that have the capacity of self-renewal, can be turned on or off to stay in activate state or quiet state, and can be expanded from a single cell to limitless numbers of cell progeny. HiPSCs from patients carry the complete genetic information of the individual, and may work as a template and model system for genome editing and correction to investigate and treat human genetic disorders and diseases. HiPSCs from patients with monogenic diseases can faithfully recapitulate the disease phenotypes in vitro when they are differentiated into disease-relevant cell types. Therefore, hiPSCs provide powerful tools and inexhaustible resources for potential applications in biomedicine.

Heart disease is the leading cause of mortality for men, women, and people of most racial and ethnic groups in the United States, and causes more deaths than all cancers combined, and costs about 219 billion each year from 2014 to 2015. About 655,000 people die of heart disease in the United States every year (1 in every 4 deaths) (https://www.cdc.gov/heartdisease/facts.htm, accessed on 5 March 2021). Coronary heart disease, or ischemic heart disease, is the most common type of heart disease, killing 365,914 people in 2017 in the United States. The major damage of coronary heart disease to human heart is the massive loss of functional cardiomyocytes in ischemic myocardium. Cardiac specification occurs very early during embryonic development, and the heart has very limited intrinsic capacity to regenerate and repair the damaged tissue, leading to high morbidity and mortality. It is clearly inadequate to reverse the damaged cardiac function following injury. To repair the damaged cardiac tissues, multiple approaches for increasing cardiomyocyte numbers in the injured adult heart have been proposed. Transplantation of in vitro derived cardiomyocytes into the injured area is an important approach for cell-based therapy. HiPSCs provide the limitless and patient-specific stem cell sources for differentiation and generation of cardiomyocyte-like cells. The American Heart Association has published a scientific statement to address the hiPSCs for cardiovascular disease modeling and precision medicine, highlighting the importance, strategies, opportunities and challenges of hiPSCs and hiPSC-CMs in cardiovascular medicine and biomedical applications [83]. Patient and disease-specific hiPSC-CMs provide unprecedented opportunities to discover new drug targets and screen compounds for cardiovascular disease, which will vastly improve the ability to test drugs efficiently, as well as tailor and titrate drug therapy for each patient [84]. Transplantation of hiPSC-CMs to the diseased heart has been intensively tested in preclinical and clinical studies, and has demonstrated to be a promising cell-based strategy for the treatment of cardiovascular diseases despite many challenges [85,86,87]. The use of patient somatic cell-reprogrammed iPSCs may help to circumvent potential immunogenicity of the transplanted iPSC-CMs.

## 7. Nanoparticles in iPSC Generation and Precision Medicine

For generation of iPSCs, somatic cells need to be reprogramed by expression of a set of transcription factor genes. The original mouse iPSCs were established by retrovirally introducing four transcription factors (c-Myc, Oct3/4, Sox2, and Klf4) into mouse fibroblasts [1]. Viral transduction is an efficient way to deliver the transgenes into the cell. However, the usage of viral transduction has safety concerns including cytotoxicity, cellular immune responses, transgene integration into host genome, generation of nonspecific mutations, oncogenesis and teratoma development in therapeutic use. Therefore, generating iPSCs through a non-viral approach is necessary and desirable. Nanoparticle-mediated transgene delivery stands out to be an efficient method for the generation of iPSCs. Using polyamidoamine dendrimer-modified magnetic nanoparticles as a delivery system, Ruan et al. developed a delivery method by transfecting 293T cells by four transcription factor genes including Oct4, Sox2, LIN28, and Nanog, and packaging plasmids such as PSPAX2 and PMD2.G, and finally the resultant supernatant was incubated with human fibroblast cells to generate iPSCs [88]. Biodegradable cationic polymer PEI-coated super paramagnetic nanoparticles were first reported to be used for direct generation of iPSCs from fibroblasts by transgene delivery and expression [89]. A similar approach using liposomal magnetofection (LMF) method for iPSC generation from mouse embryonic fibroblast cells was reported [90]. Other cation polymers such as poly-β-amino ester nanoparticles were also used to package the plasmid DNAs for their delivery to somatic cells to generate iPSCs [91,92]. Arginine-terminated generation 4 polyamidoamine (G4Arg) nanoparticles were successfully used for the delivery of a single plasmid construct carrying the four transcription factors into mouse embryonic fibroblasts to generate iPSCs [93]. Recently, several other types of nanoparticles were used for the reprograming of somatic cells to iPSCs including octadecylamine-based cationic lipid nanoparticles [9,94]. In addition, protein and peptide-based systems were used to reprogram somatic cells to iPSCs [95,96,97,98]. Nanoparticles were used for the encapsulation and conjugation of the peptides for delivery and functional integration [99,100,101,102]. The combination of peptide-based system and nanoparticles will provide a new powerful tool for iPSC generation and application.

iPSCs are undifferentiated and pluripotent cells that can be directed to differentiate into specialized cells guided by intrinsic or external cues [6,103]. Differentiation of iPSCs into specific cells depends on the expression of specific genes. The forced differentiation of iPSCs needs to be guided by external cues. Transgene delivery of inducing factors is one of the efficient ways for guiding iPSCs differentiation. However, iPSCs are difficult to transfect. Nanoparticles emerged as a novel tool for transgene delivery to iPSCs. Mesoporous silica nanoparticles (MSNs) were utilized and evaluated for the generation of iPSCs [104,105]. The study shows that FITC-conjugated MSNs (FMSNs) could be used as suitable carriers for biomolecule delivery and labeling of iPSCs. FMSNs of various surface charges could be efficiently internalized by iPSCs without causing cytotoxicity. We recently reported that magnetic nanoparticles can be successfully and efficiently used for the transfection of iPSCs and iPSC-CMs [78]. In addition, nanoparticles have also been widely used in the transgene delivery and differentiations of adult stem cells and embryonic stem cells, which have been discussed in a recent review [20].

Patient-specific iPSCs derived cells allow individualized disease modeling, drug screening, and drug testing for precision medicine. As we discussed above, nanoparticles play critical roles in generating the patient-specific iPSCs and the derived cells by providing a new transgene delivery tool. Moreover, nanoparticles can also be engineered for imaging, cell tracking, drug delivery, and tissue engineering in a more personalized manner [32,106].

## 8. Transgene Delivery to hiPSCs Using Nanoparticles

**Figure 5:**
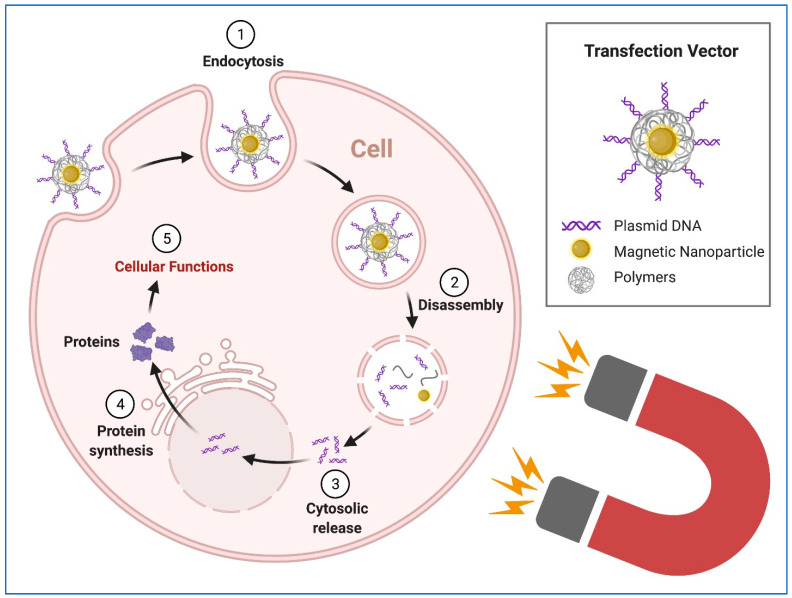
Magnetofection using magnetic nanoparticles. The plasmid DNA is associated with magnetic nanoparticles, which are directed, attracted and concentrated on the surface of cell membranes, where the endocytosis process brings the nanoparticles into the cell. Figure generated using BioRender.

HiPSCs and hiPSCs-derived cells have the potential to revolutionize regenerative, therapeutic, and precision medicine. hiPSCs represent a stable, limitless and inexhaustible source of cells for targeted differentiation into somatic cells for the downstream clinical and research applications. However, the quality-controlled culture and production of hiPSCs require highly specific culture protocols and conditions, including the defined components of culture medium, durations, and microenvironment [82,107,108]. Disruptions associated with the existing transgenic methods during the genetic modifications, such as viral transduction and chemical transfection, may introduce significant alternations to cell culture which will affect the potency, purity, consistency, and functional capacity of hiPSCs. Therefore, physical transfection methods may be better ways for the transgene delivery with minimum interference to the normal culture process. For example, electroporation has been reported as an efficient transgene delivery approach [109]. However, its clear disadvantage lies in high voltage-induced damage to cells as well as nonspecific transport of molecules in or out of the cells and disturbance to cellular homeostasis [110]. Magnetically enhanced nucleic delivery (magnetofection), first reported in 2000 with experimental evidence [53], has the potential to advance physical transgene delivery. Moreover, the superparamagnetic nanoparticles were later used for enhancing the transgene delivery [111]. Since then, magnetic nanoparticles have been widely used in the transgene delivery to the cells [53,81,112,113,114]. The principle of the magnetofection using magnetic nanoparticles is shown in Figure 5.

Magnetofection using magnetic nanoparticles in stem cells was first reported in transgene delivery to neural precursor and stem cells [115,116] and then human mesenchymal stem cells [117]. The results demonstrated an enhanced transfection with negligible toxicity. There are no adverse effects observed on stem cell proliferation and differentiation. Since then, the magnetic nanoparticles have been extensively used in transgene delivery to stem cells [118,119,120]. Therefore, magnetofection using magnetic nanoparticles offers a simple and robust approach for transgene delivery to stem cells. However, whether the iPSCs and iPSC-derived cells can be efficiently transfected or not by magnetic nanoparticles is still not tested or addressed.

**Figure 6:**
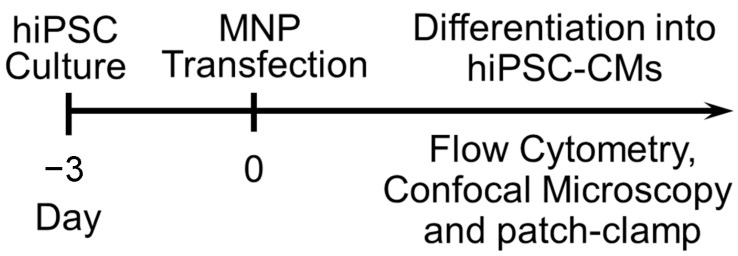
Schematic experimental protocol for iPSCs culture, biological and functional characterizations, and transfection. MNP: magnetic nanoparticle.

We proposed and tested the transfection of hiPSCs using commercially available magnetic nanoparticles [78]. A schematic representation of the experimental protocol was shown in Figure 6. The transfection was conducted following the manufacturer’s instructions (Neuromag, OZ Biosciences Inc., San Diego, CA, USA) and published methods [121,122]. The magnetic nanoparticles are positively charged, with a zeta > +30 mV in water. The size of the nanoparticles ranges from 140 to 200 nm with the majority around 160 nm, and the particle population is homogeneous. Briefly, plasmid DNAs (pIRES2-EGFP, Clontech Laboratories, Inc., Mountain View, CA, USA or a double fusion construct (an integrating vector) with green fluorescence protein (GFP) [123].) were diluted in cell culture medium, and the nanoparticles reagent was added to the culture medium containing DNA. After brief vortexing and 20-min incubation at room temperature, the medium containing the DNA/nanoparticle complexes was added to the cell culture dish. The dish was then placed on a magnetic plate and incubated in a cell culture incubator for 1, 2, and 4 h. Cells were harvested or differentiated after 24–48 h of transfection. For comparison, lipofectamine 2000 and 3000 (Thermo Fisher Scientific, Waltham, MA, USA) were used.

**Figure 7:**
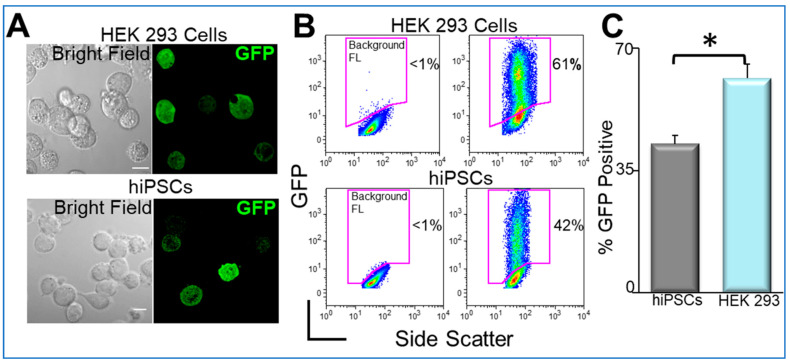
Transfected cells and the flow cytometry analysis of the transfection efficiency [78]. (**A**). Confocal laser scanning microscopic images of double fusion construct-transfected HEK 293 cells (upper) and human induced pluripotent stem cells (hiPSCs) (lower). The left panels show the corresponding bright-field images of the cells. Scale bar is 10 µm. (**B**). Flow cytometric analyses of transfection efficiencies. Magnetic nanoparticle-treated cells without green fluorescence protein (GFP) plasmids were used as control for background fluorescence (Background FL) shown in the left panel. GFP signals were detected from the GFP expression in the cells. (**C**). Summary data from D (* *p* < 0.05, *n* = 3–7).

We first tested the double fusion construct, and the confocal microscopic images of transfected cells are shown in Figure 7A. Flow cytometric analyses were used to directly quantify nanoparticle-mediated transfection in HEK 293 cells and hiPSCs (Figure 7B,C).

**Figure 8:**
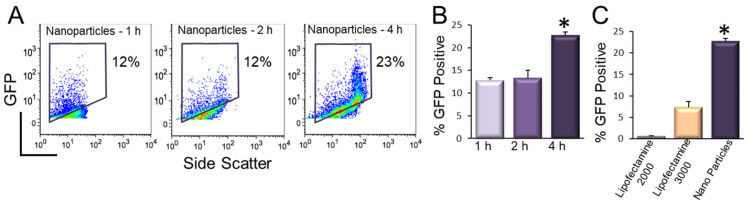
Incubation time-dependent transfection efficiency using magnetic nanoparticles [78]. (**A**). Flow cytometric analysis of pIRES2-EGFP-transfected hiPSCs using 1, 2, and 4 h of magnetofection. (**B**). Summary data from A (* *p* < 0.05, *n* = 3–7). (**C**). Comparison of the transfection efficiency of hiPSCs using pIRES2-EGFP vector and lipofectamine-2000, -3000 and nanoparticle-mediated transfections. Four hours of transfection was used for all the conditions (* *p* < 0.05, *n* = 3–7).

We further tested the transfection using a non-integrating GFP construct (pIRES2-EGFP) and optimized the time needed for magnetofection (Figure 8A,B). We note a significant increase in percentages of GFP-positive hiPSCs using 4 h of magnetofection. Importantly, there was a significant increase in the efficiency of transfection using magnetofection compared to lipofetamine-2000 and -3000 (Figure 8C).

## 9. Transfection of hiPSC-CMs Using Nanoparticles

HiPSC-CMs hold great promise for cardiac repairments and regenerations, disease modeling, drug discovery, and toxicology evaluation [84,124,125,126]. A large number of studies have refined the techniques for efficient directed-differentiation of hiPSCs into cardiomyocytes [127]. In addition, multiple studies have provided evidence for the application of hiPSC-CMs in cardiac transplantation in animal models [128]. Cardiomyocytes are terminally-differentiated and highly organized somatic cells. Transgene delivery to hiPSC-CMs by traditional chemical and viral transfection methods have been met with challenges such as low efficiency and cellular toxicity [129]. To investigate magnetic nanoparticles as a possible alternative to existing methods, we tested their ability to deliver transgenes into iPSC-CMs.

**Figure 9:**
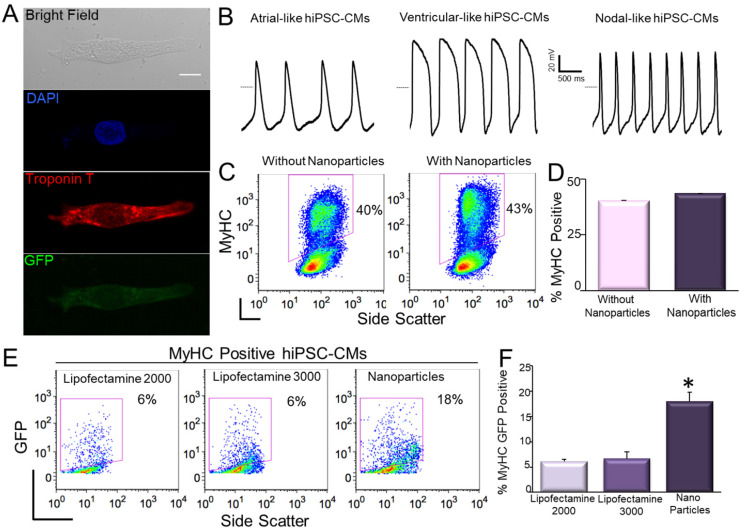
Transfection of hiPSC-CMs using magnetic nanoparticles [78]. (**A**). Confocal laser scanning microscopic images of GFP-transfected hiPSC-CM. Scale bar is 10 µm. (**B**). hiPSC-CMs exhibit spontaneous APs with ventricular-like, atrial-like, and nodal-like characteristics. The dotted line represents 0 mV. (**C**,**D**) Assessment of the efficiency of differentiation into cardiomyocytes (CMs) in control hiPSCs compared to hiPSCs transfected with double fusion construct using magnetic nanoparticles by analysis of myosin heavy chain (MyHC) positive cells. Summary data are shown in the right panels. (**E**,**F**) Comparison of the transfection efficiency in double positive hiPSC-CMs (MyHC^+^/GFP^+^) using pIRES2-EGFP vector and lipofectamine-2000, -3000, and nanoparticle-mediated magnetofections. Data were collected 4 h after transfection (* *p* < 0.05, *n* = 3).

In our study, one possible concern using magnetofection is whether the procedure may alter the differentiation efficiency of hiPSCs. Here, we first compared the differentiation efficiency between control (non-transfected) and transfected hiPSCs into cardiomyocytes (CMs). The hiPSC-CMs exhibited large beating clusters with spontaneous firing APs, consistent with populations of ventricular-like, atrial-like, and nodal-like APs (Figure 9A,B) [124]. There were no significant differences in the efficiency of differentiation into cardiomyocytes between control hiPSCs and GFP-transfected hiPSCs using magnetic naonoparticles (Figure 9C,D). We further evaluated the efficiency of magnetofection in hiPSC-CMs. By using flow cytometric analysis of both myosin heavy chain (MyHC) and GFP positive cells, we directly demonstrated that magnetofection was more efficient than lipofectamine not only in hiPSCs, but also in hiPSC-CMs (Figure 9E,F) using pIRES2-EGFP construct.

## 10. Conclusions and Perspectives

Since the original and landmark description of iPSCs, the iPSCs technology has been rapidly and extensively applied in biological research, and regenerative and precision medicine. HiPSCs provide a limitless source of human cell types needed for therapeutic, research, disease modeling, and precision medicine [4,5,125,126]. Generation and genetic modification of iPSCs are essential procedures for studies and applications of hiPSCs in biomedicine, which rely on the technical advances in the field to facilitate and promote the iPSC biology. Nanoparticles have diverse applications in biomedicine including nanoprobes and sensors, enhanced imaging, magnetic separation, drug and transgene delivery, hyperthermia and cancer therapy [19,52,53,55,56,77,81,112,114,130,131,132]. The most successful example is the recent development of SARS-CoV-2 vaccines as we discussed above. Nanoparticle-mediated gene transfer offers significant advantages over other gene transfer methods, such as high efficiency, low cytotoxicity, biodegradability, low cost, directional and distal controllability, efficient in vivo applications, and lack of immune responses [53,54,115,116,121,122]. The two cutting-edge fields of iPSCs and nanoparticles empower the cross-field development of novel techniques. The possible limitation may come from the cytotoxicity of nanoparticles, which results from the accumulation of nanoparticles in endosomes and/or vacuoles in cells [133,134]. However, iron oxide nanoparticles will be degraded through normal iron metabolism over time, although the mechanism is still not well understood [19,53]. A recent study addressed the long-term fate of the intracellular magnetic nanoparticles in mesenchymal stem cells and found substantial degradation. Stem cells may even be re-magnetized after degradation of nanoparticles, demonstrating the ability of biosynthesizing new magnetic nanoparticles from iron released in the cytosol from the degradation [135]. The natural magnetism produced by human stem cells is the key process to avoid long-term cytotoxicity of nanoparticle intake and will help to develop new tools for nanomedicine. Moreover, the distal control of nanoparticles by magnetic fields will further potentiate the in vivo application and delivery of nucleic acids to specific organs for targeted gene therapy [54]. Finally, there exist unmet needs for designing, developing, and producing nanoparticles for precision and personalized medicine [106,136,137,138,139]. Therefore, nanoparticle application in iPSCs will have unmatched potential in regenerative and personalized medicine.

## Data Availability

Not applicable.
